# Negative life events, sleep quality, and depression among older adults in Shandong Province, China: A conditional process analysis based on economic income

**DOI:** 10.1111/ggi.14914

**Published:** 2024-06-22

**Authors:** Jing Zhu, Lingzhong Xu, Long Sun, Dawei Qin

**Affiliations:** ^1^ Linyi City Hospital DRG Management and application Key Laboratory, Linyi People's Hospital Linyi China; ^2^ Centre for Health Management and Policy Research, School of Public Health Jinan China; ^3^ China National Health Commission (NHC), Key Laboratory of Health Economics and Policy Research (Shandong University) Cheeloo College of Medicine, Shandong University Jinan China; ^4^ Center for Health Economics Experiment and Public Policy Research Shandong University Jinan China

**Keywords:** depression, economic income, negative life events, older adults, sleep quality

## Abstract

**Aim:**

Negative life events have been reported as a risk factor for depression. However, the mechanism between negative life events and depression is still unclear. This study aimed to explore the mediating role of sleep quality and the moderating role of economic income in the association between negative life events and depression among older adults aged 60 years and over.

**Methods:**

A multi‐stage stratified sampling method was used to select elderly individuals over 60 years old in Shandong, China, making use of the Household Health Interview Survey (2020). In total, 3868 older adults completed the measures of negative life events, sleep quality, depression, and economic income.

**Results:**

Negative life events positively predicted depression among the elderly (proportion of direct effect, 55.12%), and poor sleep quality could mediate this association (proportion of indirect effect, 44.87%). Economic income played a moderating role in the relationship between negative life events, sleep quality, and depression (the first and second half of the mediating effect, the direct effect of negative life events on depression). Both effects were weaker among the elderly with higher economic incomes.

**Conclusions:**

Negative life events had positive effects on depression in older adults. Economic income moderated the direct effect of negative life events and the mediating effect (first and second half) of sleep quality on depression. When the elderly experience negative life events, interventions for improving their sleep quality and financial support could effectively prevent depression. **Geriatr Gerontol Int 2024; 24: 751–757**.

## Graphical abstract

This study examined the conditional process of negative life events on depression. It found that negative life events had positive effects on depression in older adults. Economic income moderated the effect of negative life events and the mediating effect of sleep quality on depression.

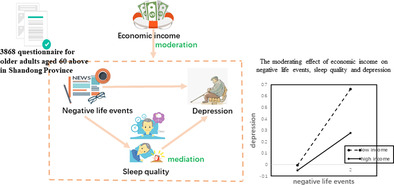

## Introduction

Population aging is a major social problem worldwide today, and China is no exception. Compared with 2010, the proportion of China's population aged over 60 years increased by 5.44%, reaching 18.7% (264 million) in 2020. It is predicted that 400 million people will be aged 65 years and over by 2050.^1^, ^2^ Therefore, China faces challenges brought on by the aging population, such as the increased risk of depression in old age, etc.^3^

Depression, the most common mental disorder among the elderly, is one of the disease burdens of an aging population.^4^ According to statistics, approximately 31.2% of older adults in China suffer from depression symptoms.^5^ And depression symptom is higher among the elderly.^6^ Depression is a severe disease, which can cause great suffering and impaired physical and mental health, thereby threatening quality of life and increasing mortality.^7^, ^8^ Therefore, the problem of depression among the elderly should not be ignored. Studies on individuals with depressive symptoms or depression have demonstrated that both conditions are associated with negative life events, poor sleep quality, and economic income.^9^, ^10^ These findings play an important role in exploring the pathogenesis of depression and preventing depression in older adults.

Geriatric depression is related to certain psychosocial factors. Negative life events are one of the psychosocial factors associated with depression.^11^, ^12^ The categories of negative life events include those related to severe illness and/or death of significant others, negative socioeconomic circumstances, negative events within relationships, and stressful life events.^9^ The prevalence of negative life events increases with increasing age.^13^ Negative life events both affect depression and increase the risk for mental disorders.^14^, ^15^ In addition, a study has shown that approximately 50% of older adults have sleep problems.^16^ Poor sleep quality is a risk factor of depression,^17^ and sleep issues frequently manifest before a new or repeated episode of severe depression.^18^, ^19^ Based on these findings, we hypothesized that negative life events positively predict depression, and sleep quality mediates the relationship between them.

The economic situation of the older adults is relatively poor because of their lower labor capacity and income.^20^ Prior studies have consistently found that socioeconomics influence factors of depression, and economic income is a component of socioeconomic.^21^, ^22^ Studies show that higher economic income is a protector against depression.^23^, ^24^ According to a study on the relationship between income and depression among Chinese residents, there is an overall downward‐trend curve between personal income and depression, and the highest point of depression appears in the lowest‐income group.^25^ Also, previous studies found that people with high socioeconomic status have less psychological stress when encountering negative life events, and thus their sleep quality is better.^26^, ^27^

**Figure 1:**
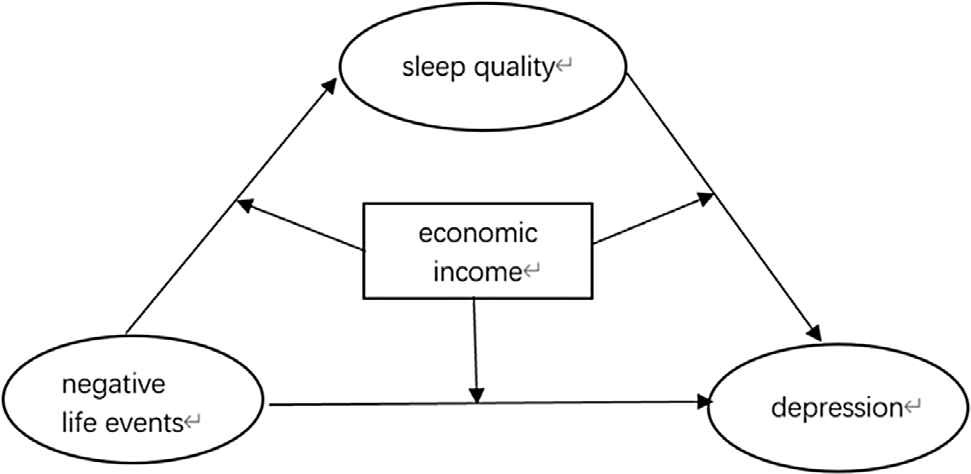
Theoretical moderated mediation model. We hypothesized that negative life events positively predict depression among older adults; that sleep quality mediates the relationship between negative life events and depression among the elderly; that the effects of negative life events on depression among older adults are moderated by economic income; and that the effects of negative life events on sleep quality and sleep quality on depression are moderated by economic income.

Based on the status of research, however, the mechanism between negative life events and depression is still unclear. Thus, we propose the following hypotheses in this study. First, negative life events positively predict depression among older adults. Second, sleep quality mediates the relationship between negative life events and depression among the elderly. Third, the effects of negative life events on depression among older adults are moderated by economic income; and the effects of negative life events on sleep quality and of sleep quality on depression are moderated by economic income. The research hypotheses were examined by a moderated mediation model (shown in Fig. 1).

## Methods

### Participants

Data were collected from the 2020 Household Health Interview Survey, which aimed to examine the health status and health service demand and utilization of residents. This cross‐sectional survey was performed in Taian City, Shandong Province, China. Stratified multi‐stage random sampling was applied to select participants from all six administrative districts (4 counties and 2 districts). The sampling method was similar to that in our previous study.^28^ In the first stage, based on the level of socioeconomic development and geographical position, using the probability proportionate to size sampling method (PPS), 3 or 4 sub‐districts/towns were randomly selected from each district or county in Taian City. In the second stage, from each town and sub‐district, 8 villages and 8 committees were selected separately (PPS), with a total of 160 villages/committees selected. Finally, an average of 50 households were randomly selected in each village/committee, making up the total sample (simple random sampling). Eligible participants were those aged 18 years or older with local household registrations. In total, 7945 households were recruited, and 7920 completed the whole survey. Finally, with a response rate of 75.8%, 7920 households consisting of 8946 individuals were included in the sample. All participants were interviewed face to face by trained interviewers in the participant's home using a paper‐based questionnaire. In this study, we included older adults aged 60 years and above. A total of 3868 older adults were included in this study.

### Measures

#### Depression

Depression among older adults was measured with the Patient Health Questionnaire‐9 (PHQ‐9).^29^ The subscale contains nine items concerning individuals’ experiences over the past 2 weeks rated on a 4‐point severity/frequency scale, ranging from 0 (did not apply to me at all) to 3 (applied to me very much, or most of the time). The total score ranges from 0 to 27. The higher the score, the more severe the depressive symptoms. In this study, Cronbach's coefficient of the PHQ‐9 was 0.825.

#### Negative life events

The negative life events in our study include death of significant others, severe illness of significant others, negative socioeconomic circumstances, negative events within relationships etc. In our research questionnaire, we used the question “Have you ever encountered any major setbacks or unexpected misfortunes in your life and work in the past two years? 1.Yes; 2. No” to assess experience of negative life events. Respondents could answer “No” (coded = 0) or “Yes” (coded = 1). This measurement has been used in previous study.^30^

#### Sleep quality

Sleep quality was measured using the Pittsburgh Sleep Quality Index (PSQI).^31^ The scale contains 19 items and seven dimensions and measures subjective sleep quality, sleep latency, sleep duration, habitual sleep efficiency, sleep disturbances, usage of sleep medication, and daytime dysfunction. Each subdimension drops score into 0, 1, 2, and 3 based on the entries it contains, and the total score of the scale is the sum of the seven dimensions, with a higher score indicating a more severe sleep problem.

#### Economic income

Participants were asked “What is your household income in 2019?” Economic income was measured by people's objective economic income, according to the analysis of the economic income sources of the elderly in China, mainly including retirement funds and pensions, support from family members, and labor income.^32^

#### Covariable

We included as covariates confounding factors that may influence depression according to previous studies. Sociodemographic characteristics included sex (male/female), residence (rural/urban), and living arrangement (living alone/living with others).^33^, ^34^

### Data analysis

All statistical analyses were conducted using SPSS 25.0. (i) We first conducted a descriptive analysis. For categorical variables, frequencies and percentages were used to present sociodemographic characteristics, and an independent *t*‐test and one‐way analysis of variance (ANOVA) were calculated for the statistical differences of depression, respectively. For continuous variables, simple linear regression analysis were calculated for the statistical differences of depression. (ii) PROCESS macro 4.0 for SPSS, developed by Hayes, was used to test mediation and the moderated mediation hypotheses.^35^ A test of the mediating role of depressive symptoms was conducted using Model 4 in PROCESS. The mediating effect was considered significant if the 95% confidence interval (CI) of the indirect effect did not contain 0. Then, we introduced economic income as the proposed moderator variable into the model and tested the moderating role of sleep quality in possible direct and indirect effects of negative life events on depression using Model 59. In PROCESS, three different levels of economic income, corresponding to Mean + 1SD, Mean, and Mean – 1SD, and conditional direct and indirect effects of negative life events on depression (via sleep quality) were examined. Before formal data processing, all variables were standardized. Statistical significance was defined as a two‐tailed *P*‐value of <0.05.

## Results

### Preliminary analysis

Table 1 reports that 3868 elderly individuals were included in the sample; the specific quantity and proportion can be seen in the table. Sex, living location, annual income, negative life events, and sleep quality were statistically significant on depression (*P* < 0.01). All the above variables were controlled as covariates in this study.

**Table 1:** Description and univariate analysis of the elderly in Shandong, China

Characteristics	*n*	%	*t/F*	*P*
Total	3868	100		
Sex			40.719	0.000
Male	1565	40.5		
Female	2303	59.5		
Residence			8.880	0.003
Rural area	2780	71.9		
Urban area	1088	28.1		
Living arrangements			35.026	0.000
Living alone	581	15.0		
Living with others	3287	85.0		
Annual income			18.356	0.000
Q1	979	25.3		
Q2	1055	27.3		
Q3	996	25.7		
Q4	838	21.7		
Negative life events			62.912	0.000
No	3693	95.5		
Yes	175	4.5		
Sleep quality	10.247 ± 7.216		−0.143	0.000
Depression	3.046 ± 3.832			

Abbreviation: Q, quarter.

### Mediation effect analysis

Table 2 shows the regression results when testing for mediation. After controlling the covariates, the total effects model shows that negative life events are positively associated with depression (*β* = 1.943, *P* < 0.001). Central to the mediation test, negative life events are positively related to sleep quality (*β* = 4.739, *P* < 0.001), and sleep quality is positively associated with depression (*β* = 0.334, *P* < 0.001). The bias‐corrected percentile bootstrap results with 5000 re‐samples show that the 95% CIs around the indirect effect (*β* = 0.0561, 95% CI, 0.3063, 0.5246) and direct effect (*β* = 0.0587, 95% CI, 0.3920, 0.6222) did not contain zero. These results confirm a partial indirect effect of negative life events on depression through the mediating variable of sleep quality. The indirect effect is 0.5071, accounting for 55.12%.

**Table 2:** Models examining the mediating role of sleep quality on negative life events and depression

Characteristics	Model 1 (depression)			Model 2 (sleep quality)			Model 3 (depression)		
	*β*	95% CI (low, up)	*P*	*β*	95% CI (low, up)	*P*	*β*	95% CI (low, up)	*P*
Negative life events (X)	3.524	[2.960, 4.088]	0.000	4.739	[3.668, 5.810]	0.000	1.943	[1.502, 2.384]	0.000
Sleep quality (M)							0.334	[0.321, 0.347]	0.000
Sex	0.996	[0.755, 1.237]	0.000	2.291	[1.833, 2.748]	0.000	0.228	[0.039, 0.417]	0.018
Residence	−0.406	[−0.668, −0.145]	0.000	0.048	[−0.449, 0.545]	0.850	−0.425	[−0.627, −0.222]	0.000
Living arrangements	−0.648	[−0.978, −0.318]	0.002	−0.677	[−1.304, −0.050]	0.034	−0.424	[−0.679, −0.168]	0.001

Characteristics	Effect	SE	95% CI		Effect percent (%)
			Low	Up	
Total effect	0.9200	0.0751	0.7727	1.0673	100
Direct effect	0.5071	0.0587	0.3920	0.6222	55.12
Indirect effect	0.4128	0.0561	0.3063	0.5246	44.87

*Note*: Bootstrap results for effect of sleep quality on depression.

Abbreviations: CI, confidence interval; M, mediating variable; X, independent variable.

### Moderated mediation effect analysis

Using the method of Muller and Judd *et al*.,^36^ the moderated mediation effect model was tested (Table 3). In model 6, negative life events had a significant positive predictive effect on depression (*β* = 0.510, *P* < 0.001), annual income had a significant negative predictive effect on depression (*β* = −0.024, *P* < 0.01), and the interaction between negative life events and annual income had a negative predictive effect on depression (*β* = −0.184, *P* < 0.01). Sleep quality had a significant positive predictive effect on depression (*β* = 0.622, *P* < 0.01). The interaction between sleep quality and annual income had a significant negative predictive effect on depression (*β* = −0.042, *P* < 0.01). The results show that sleep quality plays a partial mediating role between negative life events and depression, and economic income plays a moderating role.

**Table 3:** Mediating role of sleep quality and moderating role of income in a model examination of negative life events and depression

Characteristics	Model 4 (depression)			Model 5 (sleep quality)			Model 6 (depression)		
	*β*	95% CI	*P*	*β*	95% CI	*P*	*β*	95% CI	*P*
Negative life events (X)	0.927	[0.781, 1.074]	0.000	0.662	[0.514, 0.810]	0.000	0.510	[0.395, 0.624]	0.000
Annual income (W)	−0.060	[−0.093, −0.026]	0.000	−0.062	[−0.095, −0.028]	0.000	−0.024	[−0.615, −0.168]	0.001
Negative life events × Annual income (XW)	−0.294	[−0.440, −0.148]	0.000	−0.150	[−0.298, −0.003]	0.046	−0.184	[−0.297, −0.070]	0.002
Sleep quality (M)							0.622	[0.597, 0.646]	0.000
Sleep quality × Annual income (MW)							−0.042	[−0.067, −0.016]	0.001
Sex	0.254	[0.192, 0.317]	0.000	0.313	[0.250, 0.376]	0.000	0.057	[0.008, 0.106]	0.023
Residence	−0.061	[−0.132, 0.010]	0.091	0.050	[−0.022, 0.121]	0.176	−0.093	[−0.148, −0.037]	0.001
Living arrangements	−0.133	[−0.221, −0.046]	0.003	−0.059	[−0.147, 0.030]	0.192	−0.094	[−0.162, −0.026]	0.007

Abbreviations: CI, confidence interval; M, mediating variable; W, Moderating variable; X, independent variable.

**Figure 2:**
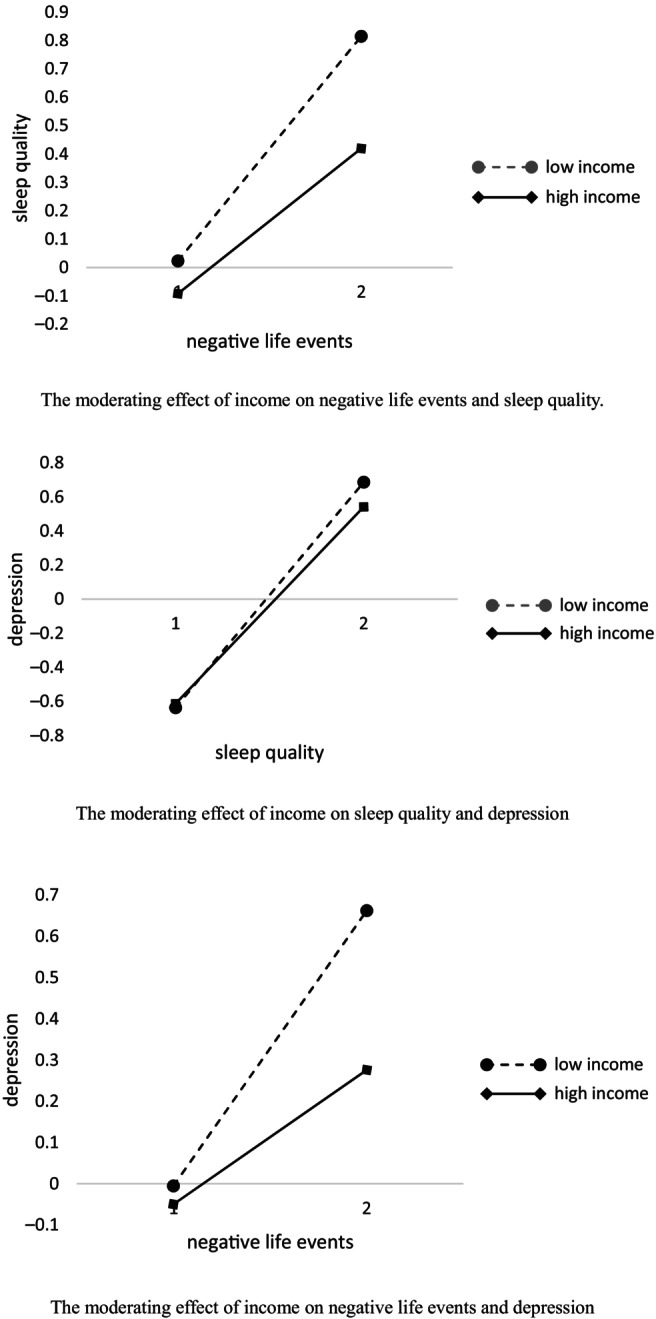
We introduced economic income as the proposed moderator variable into the model and tested the moderating role of sleep quality in possible direct and indirect effects of negative life events on depression using Model 59 in PROCESS. Three levels of economic income, corresponding to the Mean + 1SD, Mean, and Mean – 1SD, and conditional direct and indirect effects of negative life events on depression (via sleep quality) were examined. In order to further reveal the interaction effect of annual income and negative life events, as well as the interaction effect of annual income and sleep quality, the average annual income score was divided into a high‐income group and low‐income group according to the rule of adding or subtracting one standard deviation from the mean scores and drawing simple slope plots (see this figure). The three slope plots reveal the following. For the low‐income group, negative life events had a significant positive predictive effect on sleep quality. However, for the high‐income group, the positive prediction effect was weakened, and the change in the sleep quality of the elderly was less than that for the low‐income group. For the low‐income group, sleep quality had a positive effect on depression, while for the high‐income group, the positive predictive effect was weakened. For the low‐income group, negative life events had a positive predictive effect on depression, while for the high‐income group, the positive predictive effect was weakened. Compared with the low‐income group, the increase in the depression level on the elderly with high income group was significantly reduced.

In order to further reveal the interaction effect of annual income and negative life events, as well as the interaction effect of annual income and sleep quality, the average annual income score was divided into a high‐income group and a low‐income group according to the rule of adding or subtracting one standard deviation from the mean scores, drawing simple slope plots (see Fig. 2). The high‐income group weakened the effect among them.

## Discussion

This study focused on negative life events and depression among older adults in China, revealing the possible mediating and moderating mechanisms underlying the relationship between negative life events and depression via a moderated mediation model. This study found a significant positive association between negative life events and depression, and sleep quality played a partial mediation role in this association. Additionally, economic income moderated the first and second mediating effect and the direct effect of the mediation model, in which the positive effects of negative life events on depression were the highest in populations with a low income.

Negative life events positively predicted depression among the elderly, which is consistent with previous studies.^9^ One possible explanation is that the health status of the elderly deteriorates, the incidence of chronic diseases is high, and they are more likely to be disabled. An inability to take care of themselves will lead to depression.^37^, ^38^ Another explanation concerns personal relationships. The tradition of respecting, valuing, and taking care of older adults is deeply rooted in China.^39^ However, with the development of urbanization, the tremendous outflow of labor in China pushes the younger generation to live separately from their elderly parents. This living arrangement inevitably poses great challenges for adult children to provide adequate care for older parents, which results in older adults being seen as a burden.^40^ The elderly have poor psychological adaptability when facing difficulties, which leads to depression.

Furthermore, the study demonstrated that sleep quality plays a mediating role in negative life events and depression. Individuals who experienced negative life events were more likely to experience insomnia, which is consistent with existing findings.^41^ Individuals with poor sleep quality are more likely to be depressed. According to the psychological stress theory, when the elderly suffer from the death of relatives, physical illness, economic pressure, and interpersonal problems, they will experience negative emotions, which leads to sleep problems. Sleep disturbance is the core influencing factor and core symptom of depression. In epidemiological studies, insomnia in people who are not depressed has been linked to a later risk of developing depression.^42^, ^43^ A controlled, double‐blind study complemented this finding by showing that treating insomnia reduced the severity of depression.^44^ Therefore, these findings suggest that the effects of negative life events on depression can be improved by good sleep quality.

According to our moderated mediation analyses, economic income moderated the direct effect of negative life events on depression, moderated the first mediation effects of negative life events on sleep quality, and moderated the second mediation effects of sleep quality on depression. Low income is a risk factor for depression in older adults.^45^ In our study, rural elderly accounted for 71.9% of the sample. The labor ability of farmers declines with increasing age, their personal income is relatively low, and they depend more on their children.^46^ The elderly with a good economic situation can meet their daily life and medical demands.^47^ Individuals with low income and higher life stress are more likely to be depressed.^20^ The elderly with a good economic situation can obtain better medical services, and ameliorate the negative impact of negative life events. Thus, high economic income acts as a protective factor regarding health outcomes.^48^, ^49^ Higher income is also a protective factor for sleep.^50^ Higher economic income could help older adults relieve psychological stress and improve their sleep quality. Thus, it is critical to take measures to furnish financial support for individuals with negative life events and improve their sleep quality in order to decrease the negative influence of negative life events.

The public health implications of our study are that the government (China) can improve depression by enhancing economic income and improving sleep quality, which can be achieved through the following measures. First, the government must continue to increase the income of and pension benefits to the elderly, increase social financing channels, and improve medical insurance coverage in order to reduce the effect of negative life events. Second, the government needs to improve the infrastructure construction and encourage community interpersonal interactions to improve negative moods and reduce the risk of sleep disorders and depression among the elderly.

This study has some limitations, however. The cross‐sectional study design limits our ability to claim any causal relationships among negative life events, sleep quality, and depression. Future longitudinal studies are needed to further investigate the impact of negative life events and sleep quality on depression among the elderly from a developmental perspective. Furthermore, the data were collected through self‐reporting, so recall bias may exist, for example regarding experiences of negative life events.

## Conclusion

The results from our study support previous findings that elderly individuals who have experienced negative life events are more likely to suffer from depression. The underlying mechanisms between negative life events and depression were as follows. Sleep quality partially mediated the relationship between negative life events and depression among the elderly. Economic income had a moderating effect: higher economic income weakened the effect of negative life events on poor sleep quality and depression, and weakened the effect of poor sleep quality on depression. Our findings have public health implications for the prevention of and intervention in the impact of negative life events on depression, that active intervention in sleep quality and financial support may play an important role in resolving depression among older adults.

## Funding information

This research was supported by the National Natural Science Foundation of China (71974118 and 72204145), “Feasibility study of disease category management based on big data in improving the structure of hospital disease category” (NHC‐HEPR2019004), and Science and Technology Development Project of Affiliated Hospital of Weifang Medical College (2023FYZ003).

## Disclosure statement

The authors declare no conflict of interest.

## Author contributions

All the authors participated in this research. Data analysis and manuscript writing were all completed by JZ. Data were collected by the graduate team of LX. The concept analysis and revision of the manuscript was done by JZ, LX, and DQ. All authors contributed to the paper and approved the submitted version.

## Ethics statement

The studies involving human participants were reviewed and approved by the Ethical Committee of the Centre for Health Management and Policy Research, Shandong University (approval number: LL20191220). The patients/participants provided their written informed consent to participate in this study.

## Consent for publication

All the authors agreed to publish the paper.

## Data Availability

The datasets supporting the conclusions of this article are available from the corresponding author on reasonable request.
